# Carp-Derived Antioxidant Peptides and Hydrolysates: Biological Effects and Potential Applications in Health and Food

**DOI:** 10.3390/antiox14091095

**Published:** 2025-09-08

**Authors:** Fai-Chu Wong, Wen-Jie Ng, Ai-Lin Ooi, Fui-Fui Lem, Tsun-Thai Chai

**Affiliations:** 1 Department of Chemical Science, Faculty of Science, Universiti Tunku Abdul Rahman, Kampar 31900, Malaysia; wongfc@utar.edu.my; 2 Center for Agriculture and Food Research, Universiti Tunku Abdul Rahman, Kampar 31900, Malaysia; ooial@utar.edu.my; 3 Department of Allied Health Sciences, Faculty of Science, Universiti Tunku Abdul Rahman, Kampar 31900, Malaysia; ngwj@utar.edu.my; 4 Centre for Biomedical and Nutrition Research, Universiti Tunku Abdul Rahman, Kampar 31900, Malaysia; 5 Department of Agricultural and Food Science, Faculty of Science, Universiti Tunku Abdul Rahman, Jalan Universiti, Bandar Barat, Kampar 31900, Malaysia; 6 Scientific Research Society of Sabah, Lot 2, Ground Floor, Block A, Lorong Grace Square, Jalan Pantai Sembulan, Kota Kinabalu 88100, Malaysia; seres072023@gmail.com

**Keywords:** antioxidant peptide, bioactivity, by-product, carp, enzymatic hydrolysis, food preservation, nutraceutical, oxidative stress, protein hydrolysate, valorization

## Abstract

Oxidative stress is a factor implicated in chronic diseases and aging, motivating the search for natural antioxidants. Over the past ten years, food-derived peptides have been recognized as potent antioxidants. Carp, a globally farmed fish, is a protein-rich raw material for producing antioxidant peptides and hydrolysates. This review summarizes the current knowledge on these antioxidant peptides and hydrolysates, including their production, bioactivity, and applications. We discuss how enzymatic hydrolysis of carp by-products (e.g., skin, scales, and swim bladders) represents a strategy for waste valorization. Cellular and in vivo findings demonstrate the effectiveness of carp peptides and hydrolysates in tackling oxidative stress by reducing reactive oxygen species and enhancing cellular antioxidant enzymes. In addition to their antioxidant properties, these peptides and hydrolysates also possess anti-inflammatory, anti-melanogenic, and wound-healing properties. Potential applications of carp peptides and hydrolysates include their use as natural food preservatives and as active ingredients for skincare, nutraceuticals, and sports nutrition. Future research should focus on validating the in vivo bioavailability and assessing the long-term safety of carp peptides and hydrolysates to support their potential application in health. Carp-derived peptides are a valuable resource for developing functional foods and health products, which can contribute to a more sustainable food industry.

## 1. Introduction

Oxidative stress is a consequence of an imbalance between reactive oxygen species (ROS) production and antioxidant defenses in the body. It has been implicated in the pathologies of chronic diseases and the process of aging [1,2]. Hence, there has been continuous interest in the global research community to explore the therapeutic and disease-preventive potential of antioxidants [3]. Recent advances in the development of antioxidant therapies for disease management have been comprehensively reviewed [4,5,6]. Moreover, the applications of antioxidants extend beyond therapeutic use and functional food formulation, encompassing skincare products [7] and food preservatives [8,9].

Over the past decade, antioxidant peptides derived from food sources and their processing by-products have emerged as promising antioxidant agents. They show potential for applications in human health, cosmetics, and food product preservation [10,11,12]. Food-derived antioxidant peptides typically consist of 2–20 amino acids and may exert antioxidant action either in vitro or in vivo. Such peptides may directly scavenge ROS, intercept free radical chain reactions, chelate metal ions, and/or regulate gene and protein expression of oxidative and antioxidant enzymes in cells and organisms [11,12,13].

Both marine and freshwater fishes are well-recognized for their potential to yield potent antioxidant peptides and hydrolysates with therapeutic applications, as well as for use as natural food preservatives, nutraceuticals, and cosmetic ingredients. Freshwater fish, such as carp, catfish, and tilapia, offer distinct advantages over marine fish due to their cost-effective production [14,15,16,17], higher aquaculture yields [18,19], and sustainability as widely farmed species [14,18]. Notably, as one of the most farmed freshwater fish species worldwide [18], carp represent a sustainable and readily available source of antioxidant peptides. To the best of our knowledge, no review has specifically addressed carp as a source of antioxidant peptides.

This review aims to provide an overview of carp-derived antioxidant peptides and hydrolysates, focusing on their biological effects and potential applications in health and food systems. Specifically, this review will: (1) summarize the diversity, aquaculture, and proximate composition of commercial carp species; (2) examine enzyme-assisted production of carp hydrolysates and strategies for the purification and identification of antioxidant peptides; (3) analyze cellular and in vivo evidence of the antioxidant activities of carp peptides and hydrolysates; (4) explore the potential of carp peptides and hydrolysates as food preservatives; and (5) discuss potential applications of carp peptides and hydrolysates in human health. In addition, current gaps and future research directions will be presented. This review is based on a comprehensive literature search of the PubMed and Scopus databases, using the following keywords: “carp”, “antioxidant”, “peptide”, and “hydrolysate”. The search covered publications from 2014 to the present. By comprehensively analyzing current knowledge, this review aims to provide insights into the potential applications of carp-derived peptides and hydrolysates in human health, cosmetics, and food preservation.

## 2. Carp as a Bioresource

### 2.1. Species Diversity and Global Production

**Figure 1:**
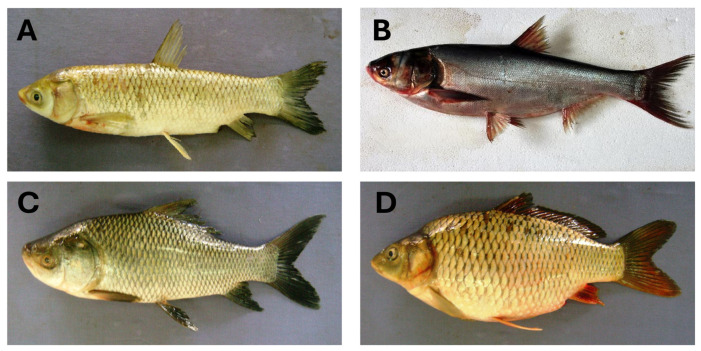
Major cultured species of carp. (**A**) Grass carp (*Ctenopharyngodon idella*), (**B**) silver carp (*Hypophthalmichthys molitrix*), (**C**) catla (*Labeo catla*), and (**D**) common carp (*Cyprinus carpio*). (Photo credits: (**A**,**C**,**D**)—Hamid Badar Osmany, FishBase; (**B**)—G.N. Hirimuthugoda, from FishBase.

Carp, belonging to the Cyprinidae family, is the largest family of freshwater fishes globally with around 2400 species and 220 genera [20,21]. Global freshwater fish production relies primarily on carp. In 2022, cultured carp production was reported in 104 countries or regions [22]. While around 51 species of carp are cultured, most commercial production relies on only a few key species (Table 1). Major cultured species include grass carp (*Ctenopharyngodon idella*), silver carp (*Hypophthalmichthys molitrix*), catla (*Labeo catla*), common carp (*Cyprinus carpio*) (Figure 1), and bighead carp (*Hypophthalmichthys nobilis*) [23]. These species are cultured worldwide, particularly in Central and Eastern Europe and Asian countries, such as China, Japan, Bangladesh, India, and Pakistan [24]. Most carp are consumed domestically. In 2022, 31.7 million tons of carp were produced globally, accounting for 52% of inland aquaculture fish production [22]. Among the cultured species, grass carp was the leading species in 2022, followed by silver carp, catla, common carp, and bighead carp (Table 1). The production of these carp species has steadily increased over the past two decades, indicating their growing importance in global aquaculture.

### 2.2. Aquaculture Practices

Carp species are known for their diverse diets and adaptability, making them suitable for various aquaculture practices. They feed at all trophic levels, including higher plants, aquatic vegetation, phytoplankton, zooplankton, zoobenthos, detritus-associated bacteria, and even other fish. Some species are highly opportunistic, varying their diets by season and environment [24]. As a result, polyculture systems, in which different carp species are raised together, effectively optimize resource utilization and sustainability. An example of a polyculture system is one where plankton feeders, such as silver carp, bighead carp, and catla, coexist with herbivorous species like grass carp and rohu (*Labeo rohita*) [25,26].

Among the numerous carp species, grass carp, silver carp, and common carp stand out due to their dominance in global aquaculture production (Table 1), and their adaptability to diverse aquaculture systems across Asia and Europe [27,28,29,30]. While catla also ranks high in production, its cultivation takes place primarily in South Asia [14,19,31]. Grass carp, native to China, exhibits notable reproductive capabilities, rapid growth rates, and high nutritional value. It provides inexpensive and high-quality animal protein for consumers. Grass carp tolerates a wide temperature range of 0–38 °C, salinities up to 10 ppt, and oxygen levels as low as 0.5 ppm. It feeds on higher aquatic plants, submerged grasses, detritus, and invertebrates [32,33]. Silver carp, a surface feeder, consumes phytoplankton and zooplankton in shallow (0.5–1.0 m) and warm (>21 °C) waters. It has a longer digestive canal compared to grass carp; hence, its digestion and utilization of feed are relatively efficient. The growth rate of silver carp depends on stocking density, natural food availability, and the type of supplied feed. Growth is also influenced by competition with other species in polyculture, feed conversion rate, and environmental conditions [34]. Common carp, a globally significant aquaculture species, adapts to diverse ecological conditions. It thrives within a temperature range of 3–35 °C and can adjust its diet and behavior when food is scarce [35,36]. Together, the adaptability and productivity of grass carp, silver carp, and common carp underscore their significant role in sustainable aquaculture and their importance as a protein-rich resource.

### 2.3. Protein Composition and Suitability for Antioxidant Peptide Production

All carp tissues are protein-rich and can serve as a valuable source of animal protein. The crude protein content in carp varies by species and body part. Specifically, grass carp has a crude protein content of 11.5–20.3% [27,37,38,39,40,41,42], silver carp 15.8–17.6% [27,41], and common carp 15.2–19.4% [41,43,44,45,46,47,48,49]. In bighead carp, protein levels differ by body part, with 17.5% in the dorsal muscle and 12.5% in the belly muscle [50]. To evaluate the potential of carp as a candidate for antioxidant peptide production, we compared its protein content with that of two widely farmed aquaculture species. Nile tilapia (*Oreochromis niloticus*) has a protein content of 14.8–18.8% [51,52,53,54], whereas African catfish (*Clarias gariepinus*) contains 15.2–15.4% protein [54,55]. These comparisons indicate that carp often matches or surpasses tilapia and catfish in protein content.

Detailed amino acid compositions for specific carp species and tissue types are presented in Table 2. Carp muscle generally exhibits a rich profile of proteinogenic amino acids, particularly a notable abundance of aromatic and hydrophobic residues. For example, phenylalanine, an aromatic amino acid, is abundant in grass carp (7.41%) and silver carp (7.02%). Tyrosine, another aromatic amino acid, is also present across the evaluated species. In addition, glycine (15.83%) and proline (8.39%), both hydrophobic amino acids, are abundant in common carp. The abundance of aromatic and hydrophobic amino acids is desirable, as their presence in peptides is often associated with antioxidant activity [15]. The mechanisms by which these residues contribute to the antioxidant efficacy of peptides will be elaborated in a subsequent section. Taken together, the high protein content of carp, its wide availability [18], its demonstrated potential for peptide production (see Section 3 and Section 4 below), and its rich composition of aromatic and hydrophobic amino acids, underscore its suitability as a raw material for antioxidant peptide production.

## 3. Production of Antioxidant Hydrolysates and Discovery of Peptides

### 3.1. Typical Workflow

**Figure 2:**
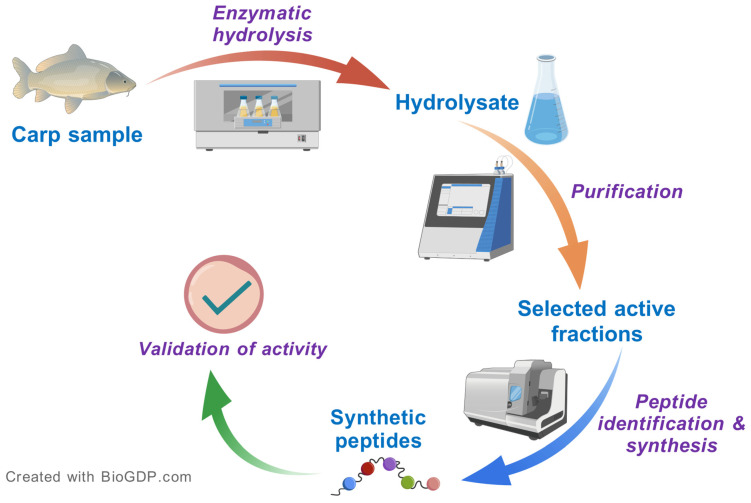
A typical approach for isolating and identifying antioxidant peptides from carp protein hydrolysates. Created with BioGDP.com [56].

The discovery of antioxidant peptides from carp typically begins with the enzymatic hydrolysis of various fish tissues. This resulting hydrolysate is then subjected to preliminary antioxidant activity screening using in vitro assays. Guided by these assays, active fractions are further purified using various chromatographic techniques. Following purification, the most potent fractions are analyzed using mass spectrometry for peptide identification. Once candidate peptides are identified, they are chemically synthesized and tested to validate their antioxidant activity. This sequential approach, summarized in Figure 2, outlines the systematic workflow commonly employed in the identification of antioxidant peptides from carp.

### 3.2. Raw Materials and Valorization

Various tissues of nine carp species have been explored as protein sources for enzyme-assisted production of antioxidant hydrolysates (Table 3), with the three most studied species being silver, grass, and common carps. Relatively underexplored species include catla, rohu, mrigal (*Cirrhinus mrigala*), crucian carp (*Carassius auratus*), and black carp (*Mylopharyngodon piceus*). There has been a growing effort to valorize low-value by-products of carp processing as raw materials for antioxidant hydrolysate and peptide production. Such a development favors waste reduction and adds value to carp processing by-products. Examples of such by-products are skin, scales, and swim bladders of various carp species. Interestingly, besides solid raw materials, liquid effluent from industrial carp processing in the form of cooking juice has been used [57]. Using these by-products for peptide production is likely more cost-effective than using carp muscle intended for filets, potentially leading to more affordable health products for consumers. Nonetheless, caution is needed when using carp gills, as they bioaccumulate higher levels of heavy metals compared to muscle tissue [58,59]. This may complicate their use in hydrolysate production as pretreatment for heavy metal removal may be required.

### 3.3. Proteases Used and Simulated Gastrointestinal Digestion

Enzymatic hydrolysis is known to produce fish protein hydrolysates with diverse health-promoting potential and high nutritional value [129]. Compared to acid and alkali hydrolysis methods, it requires milder pH and temperature conditions, besides being easier to control. It also avoids issues such as loss or destruction of amino acids and the issue of racemization [130,131]. Thus, it is a widely adopted strategy for generating antioxidant peptides and hydrolysates from carp. Among commercial proteases, Alcalase is the most widely used. It has been applied primarily on its own but occasionally combined with another protease to generate antioxidant hydrolysates from various tissue samples of silver carp, grass carp, common carp, bighead carp, catla, and rohu (Table 3).

Several studies used combinations of commercial proteases, e.g., pepsin and pancreatin [57,104,126], pepsin and trypsin [82], as well as pepsin, trypsin, and chymotrypsin [85] to simulate gastrointestinal (GI) digestion of carp samples. Such a strategy mimics natural human digestion, enabling the prediction of antioxidant peptides that may be released from digested carp proteins. For this reason, Zhang et al. [57] subjected crucian carp cooking juice to simulated GI digestion. This generated a hydrolysate containing approximately 61% low-molecular-weight (LMW) peptides (<2.4 kDa), dominated by dipeptides and free amino acids, particularly tyrosine. The LMW peptide fractions were shown to exhibit antioxidant properties, including free radical scavenging and iron chelating activities. Unlike most studies that simulated GI digestion in vitro by using commercial digestive proteases of non-human origin, Borawska et al. [98] adopted an ex vivo approach by hydrolyzing common carp muscle with human gastric and duodenal juices. Such an ex vivo approach is notable as it is more physiologically relevant and representative of the actual human digestion in comparison with the static in vitro digestion performed by others by using non-human GI enzymes [132,133]. In the study, the duodenal juice hydrolyzed carp muscle proteins more efficiently compared to the gastric juices. In line with this, the hydrolysate generated by duodenal juice exhibited stronger antioxidant activities than that produced by human gastric juice, as determined by the 2,2’-azino-bis(3-ethylbenzothiazoline-6-sulfonic acid) (ABTS) radical scavenging assay. The study proposed that carp muscle proteins are a potential source of antioxidant peptides that can be released from carp muscle proteins during human digestion [98].

### 3.4. Novel Microbial Proteases and Their Applications

Several studies have used novel microbial proteases derived from microbes in extreme habitats to prepare antioxidant protein hydrolysates from carp tissues. Such proteases can achieve levels of peptide bond hydrolysis or peptide production comparable to those obtained using commercial proteases. For example, a serine protease from cold-adapted *Planococcus* bacterium generated a silver carp muscle hydrolysate consisting of approximately 99% of LMW peptides (<1000 Da) [68]. On the other hand, protease EK4-1 extracted from the Arctic bacterium *Mesonia algae* K4-1 maintained high proteolytic activity on grass carp skin, achieving an 80% degree of hydrolysis at a low temperature of 20 °C [89]. In contrast, a protease extracted from the halophilic bacterium *Nesterenkonia* sp. was less efficient than Alcalase in hydrolyzing proteins from common carp muscles. Nonetheless, it still produced peptide fractions with free radical scavenging activity comparable to those from Alcalase hydrolysis [96].

### 3.5. Purification and Validation Pipeline

In general, following enzymatic hydrolysis, peptides are typically purified using chromatographic techniques and identified through mass spectrometric analyses. However, not all studies have taken the additional crucial step of synthesizing the identified peptide sequences and validating their antioxidant activity. Several studies have identified peptide sequences from hydrolysates with demonstrated antioxidant activity. Examples include: five peptides identified from papain-hydrolyzed bones of silver carp [76]; 178 peptide sequences identified from crucian carp swim bladder subjected to simulated GI digestion [126]; and 84 peptides identified from the neutral protease-hydrolyzed gill proteins of the bighead carp [114]. However, without validation, it remains uncertain whether each such peptide is truly an effective antioxidant, or whether it contributed to the effects observed in the hydrolysate from which it was derived. In Table 4, only selected studies that validated the identified antioxidant peptides are presented. While different carp species have been investigated, grass carp has attracted greater research interest, with more validated antioxidant peptides discovered (Table 4).

## 4. Antioxidant and Other Bioactivities of Carp-Derived Peptides and Hydrolysates

### 4.1. Cellular Models and Stressors

Various in vitro cell lines have been employed to investigate the antioxidant efficacy of carp-derived peptides and hydrolysates, focusing on their ability to inhibit ROS generation, prevent oxidative damage, and enhance antioxidant enzyme production. Both normal and cancerous human cell lines have been evaluated in these studies. The normal human cell lines examined include keratinocytes [76] and umbilical vein endothelial cells [67]. The cancerous human cell lines include intestinal carcinoma cells [65,135], leukemia monocytic cells [76], melanoma cells [76], neuroblastoma cells [86], and liver cancer cells [92]. Non-human cell lines used in such research include mouse fibroblasts [75,76], mouse hippocampal neuronal cells [109], murine melanoma cells [136], rat intestinal epithelial cells [126], and a fusion cell line derived from rat embryonic mesencephalon and mouse neuroblastoma [86]. These cellular models are used to investigate the antioxidant mechanisms of carp-derived peptides and hydrolysates, offering insights into their skin-protective applications and, to a more limited extent, their neuroprotective and anti-inflammatory potentials.

Various agents have been used to develop oxidatively stressed cell models for the characterization of carp-derived antioxidant peptides and hydrolysates. These include 2,2′-azobis(2-amidinopropane) dihydrochloride (AAPH) [65], hydrogen peroxide (H_2_O_2_) [66,126,137], tert-butyl hydroperoxide (*t*-BHP) [75,76], lipopolysaccharide (LPS) [76], oxidized low-density lipoprotein [67], 6-hydroxydopamine (6-OHDA) [86], and ultraviolet B (UVB) radiation [76]. AAPH is an azo compound that generates free radicals in cells [138]. H_2_O_2_ is a ROS that can be converted into highly reactive hydroxyl radicals [139]. *t*-BHP induces oxidative stress by depleting the antioxidant glutathione (GSH) and is commonly used to induce cellular senescence in skin cells [140]. 6-OHDA readily undergoes oxidation, generating reactive and cytotoxic products such as H_2_O_2_, para-quinones, and superoxide anion radicals [141]. Ultraviolet radiation can lead to the generation of ROS and reactive nitrogen species in cells and tissues [142].

### 4.2. Cellular Antioxidant Effects of Hydrolysates

Protein hydrolysates prepared from different carp tissues have been shown to protect cells against oxidative stress, leading to increased cell viability and reduced apoptosis. The hydrolysates could dose-dependently reduce intracellular ROS production [65,66,67,75,76,92,109,126]. Malondialdehyde (MDA) content, a key marker of lipid peroxidation, is reduced after oxidatively stressed cells are treated with carp hydrolysates [11,76,126,137]. They can also preserve mitochondrial integrity by enhancing the mitochondrial transmembrane potential (MMP) and protect cell membranes from oxidative injury, as indicated by a reduction in lactate dehydrogenase (LDH) release [67].

Carp-derived hydrolysates have also been shown to restore cellular antioxidant defenses by enhancing the activities of endogenous enzymatic antioxidants, such as superoxide dismutase (SOD), glutathione peroxidase (GPx), and catalase (CAT), in cells undergoing oxidative stress [66,67,76,126,137]. SOD converts superoxide anion radicals to H_2_O_2_, while CAT catalyzes the decomposition of H_2_O_2_ to water and oxygen; GPx utilizes GSH to degrade H_2_O_2_ into water while oxidizing GSH to glutathione disulfide (GSSG) [143]. Beyond these antioxidant functions, some hydrolysates have also been shown to exhibit anti-inflammatory, anti-melanogenic, neuroprotective, and tissue repair properties. A detailed summary of all these cellular effects is presented in Table 5.

The studies summarized in Table 5 show that various carp species are sources of effective antioxidant hydrolysates. Among these, silver carp is the most frequently investigated species, with its hydrolysates exhibiting diverse bioactivities across different cellular models. Notably, hydrolysates produced from carp by-products are also shown to be highly potent. Findings presented in Table 5 reveal that by-products such as skin, bones, and scales often yield hydrolysates that are comparable or superior to those from carp muscle in terms of multifunctionality, substantiating the value of valorizing carp by-products.

Two related studies by Wang et al. [66,67] investigated the effects of simulated GI digestion on antioxidant hydrolysates from silver carp muscle, initially produced using Alcalase and papain. The GI-digested hydrolysates not only retained their protective effects against oxidatively stressed cells but also enhanced cellular antioxidant enzyme activities. This improvement was attributed to an increase in the proportion of LMW peptides following simulated GI digestion.

### 4.3. Cellular Antioxidant Effects of Peptides

**Figure 3:**
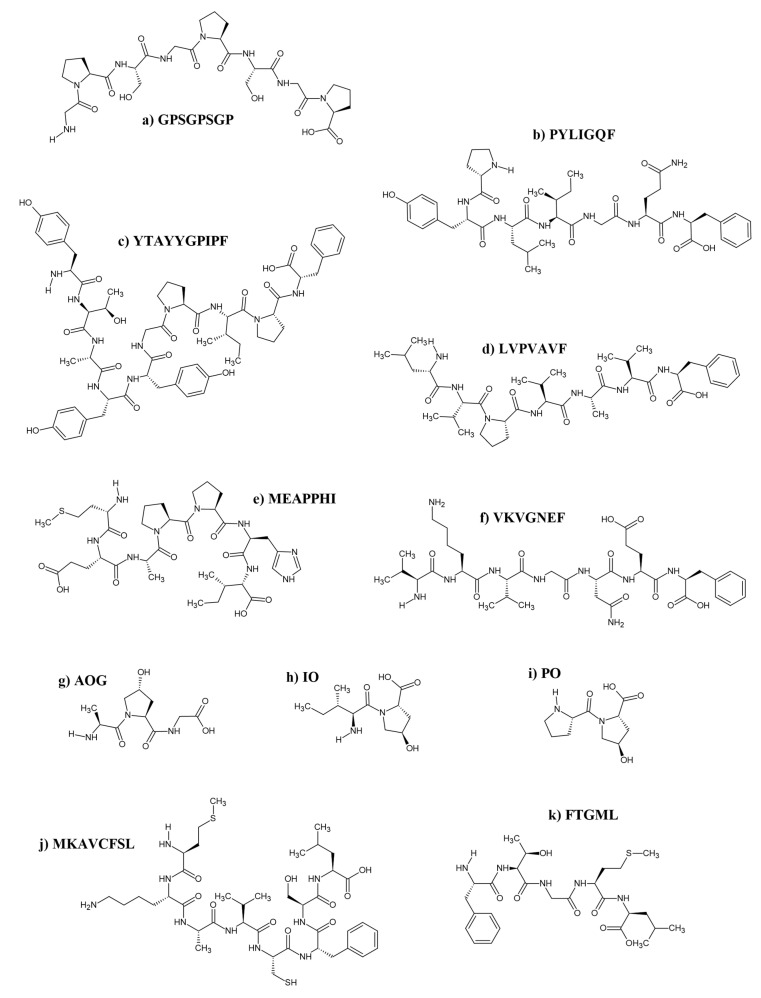
Structures of validated antioxidant peptides derived from the silver carp (**a**–**i**), bighead carp (**j**), and grass carp (**k**). Peptides were drawn using the ACD/ChemSketch freeware (version 2022.1.0, Advanced Chemistry Development, Inc. (ACD/Labs), Toronto, ON, Canada, www.acdlabs.com).

Table 6 provides a summary of 27 unique peptide sequences derived from carp protein hydrolysates. These peptides exhibit diverse cellular effects, including antioxidant enhancement, cellular protection, anti-melanogenic activity, modulation of signaling pathways, and support for skin regeneration. Specifically, in addition to attenuating ROS production, these peptides can enhance endogenous antioxidant enzyme activities [67,113,144], inhibit melanin synthesis [137], and promote collagen production in cellular models [72]. Figure 3 displays the two-dimensional structures of selected peptides listed in Table 6.

A recent study identified three antioxidant peptides, GPSGPSGP, PYLIGQF, and YTAYYGPIPF (Figure 3), from silver carp steak through dual enzymatic hydrolysis with alkaline protease and flavor enzyme [144]. Notably, GPSGPSGP and PYLIGQF contain several hydrophobic amino acids, such as phenylalanine and proline, at their N- and/or C-termini. These hydrophobic residues, particularly phenylalanine, are known to act as hydrogen donors to scavenge free radicals, consistent with findings that the presence of terminal hydrophobic amino acids is correlated with antioxidant activity [146]. Similarly, YTAYYGPIPF is characterized by a tyrosine residue at its N-terminus [144]. This residue provides phenolic hydroxyl groups capable of stabilizing free radicals [147]. Through molecular docking analysis, the study predicted that these peptides can effectively occupy the Nrf2 binding site in the Kelch domain of Keap1, thus suggesting that they may inhibit Keap1-Nrf2 interactions in cells. This predicted mechanism may have activated the Keap1-Nrf2-antioxidant response element pathway, leading to the authors’ observation of increased SOD activity and reduced MDA and ROS levels in oxidatively stressed liver cancer cells [144].

### 4.4. Other Bioactivities of Carp Peptides and Hydrolysates

Carp-derived peptides and hydrolysates exhibit multifunctional bioactivities beyond their primary antioxidant effects. These include significant wound-healing activity, anti-melanogenic effects, and, to a lesser extent, anti-inflammatory properties. Such diverse effects may result from their antioxidant action or other distinct molecular mechanisms.

#### 4.4.1. Wound Healing and Skin Regeneration

A hydrolysate derived from silver carp skin collagen has demonstrated wound-healing properties [75]. By preventing excessive ROS formation in fibroblast cells, the hydrolysate protected them against oxidative damage. Moreover, it stimulated fibroblast proliferation and promoted cell migration in an in vitro scratch-wound model, suggesting its beneficial effects on tissue repair and skin wound healing [75].

Grigore-Gurgu et al. [77] combined papain hydrolysate derived from phytophagous carp bone tissue with yellow onion skins to develop a new bioactive formulation. The microencapsulated samples restored the viability of H_2_O_2_-treated mouse fibroblasts to levels comparable to those of untreated cells. The treated fibroblasts also retained a normal fusiform morphology with clear cytoplasm. However, the bioactivities of these microencapsulated samples were not compared with those of the hydrolysate or onion skin extract alone. Thus, whether any synergistic interaction between peptides and flavonoids occurred remains uncertain. Moreover, their effects on ROS formation and the endogenous antioxidant system warrant further study.

Specific carp antioxidant peptides that directly promote skin repair have also been reported. Liu et al. [72] extracted serum from rats that had been orally administered collagen hydrolysates produced from silver carp skin. Out of eight hydroxyproline-containing peptides identified in the serum, two dipeptides (IO and PO) and one tripeptide (AOG) (Figure 3) exhibited the most prominent photoprotective and reparative effects in ultraviolet A-induced skin fibroblasts [72] (Table 6). IO and AOG enhanced procollagen production, whereas IO and PO increased hyaluronic acid secretion, thereby contributing to skin regeneration. Furthermore, AOG downregulated the expression of a stress-related transcription factor, indicating a role in mitigating collagen degradation [72]. These findings provide insight into the mechanism underlying the anti-photoaging effect of these peptides on the skin.

#### 4.4.2. Anti-Melanogenic Effects

FTGML (Figure 3), a grass carp scale-derived peptide, exhibits antioxidant and anti-melanogenic properties [137]. It reduces ROS and MDA levels, enhances antioxidant enzyme activities, and regulates GSH levels to maintain a reductive cellular environment [137]. It also decreases melanin content by inhibiting tyrosinase activity. Mechanistically, FTGML downregulates key signaling pathways involved in melanogenesis, including the cyclic adenosine 3’, 5’-monophosphate-mediated cascade and the phosphatidylinositol 3-kinase/protein kinase B pathways (Table 6). These actions ultimately suppress the expression of the microphthalmia-associated transcription factor and melanin synthesis [137].

In addition to FTGML, other antioxidant peptides derived from carp have also shown anti-melanogenic properties. For example, a selenocysteine-modified peptide derived from silver carp scales reduced cellular oxidative stress and inhibited melanin-related gene expression in B16 melanoma cells. These effects were achieved partly through the modulation of key signaling pathways, highlighting the peptide’s potential as a melanin inhibitor [145] (Table 6).

Beyond specific peptides, carp hydrolysates have been demonstrated to suppress melanin production in UVB-irradiated melanoma cells through the inhibition of tyrosinase activity, a key enzyme in melanogenesis [76,136]. Although further research is required to identify the specific peptides responsible for these effects, this inhibition of UVB-induced pigmentation may be beneficial for photoprotection, especially in the cosmeceutical industry.

#### 4.4.3. Anti-Inflammatory Activity

Carp-derived hydrolysates show anti-inflammatory effects primarily by suppressing the production of pro-inflammatory cytokines. For instance, silver carp bone hydrolysate lowered the levels of tumor necrosis factor-alpha (TNF-α) and interleukin-1 beta (IL-1β), two key mediators of the inflammatory response in LPS-treated human monocytic leukemia cells [76] (Table 5). Similarly, hydrolysates produced from the swim bladder of crucian carp suppressed the expression of TNF-α, interleukin 6, and IL-1β in H_2_O_2_-treated rat intestinal epithelial cells [126]. These findings support the potential of carp-derived hydrolysates as anti-inflammatory agents.

### 4.5. Bioavailability

Bioavailability refers to the proportion of an ingested substance that reaches the systemic circulation in an active form, encompassing gastrointestinal stability, absorption, first-pass metabolism, and distribution [148,149]. Orally administered protein hydrolysates and peptides must resist GI degradation and be absorbed to exert systemic antioxidant effects. During digestion, these compounds are exposed to low pH in the stomach and digestive enzymes, which break down the peptides into smaller fragments [149]. This controlled proteolysis is essential for releasing bioactive fragments from food matrix into the gut lumen in a form suitable for absorption, a process known as bioaccessibility [150]. Once released, these peptides may undergo further modification by intestinal peptidases, be transported across the intestinal wall via various pathways, and undergo subsequent metabolic transformations [151,152]. Thus, understanding the factors that influence both bioaccessibility and intestinal absorption is essential for evaluating the true bioavailability and physiological efficacy of antioxidant protein hydrolysates and peptides.

Several recent studies have examined carp-derived antioxidant peptides and hydrolysates using simulated GI digestion and intestinal transport assays. Wang et al. [66,67] characterized the absorption phase of papain- and Alcalase-derived silver carp hydrolysates through simulated GI digestion followed by Caco-2 cell transport assays. Caco-2 cells are a human intestinal epithelial cell line widely used for modeling intestinal absorption in vitro. When these GI-digested hydrolysates were tested for absorption through a Caco-2 cell monolayer, between two and three of the hydrolysate fractions were successfully transported. Although the ROS and MDA inhibitory activities of these transported peptides were reduced, their overall antioxidant activity remained high. This outcome indicates that antioxidant peptides in the GI-digested hydrolysates can be absorbed by intestinal cells while retaining their bioactivity [66,67]. These findings provide strong in vitro evidence for a crucial step towards achieving systemic bioavailability.

Wang et al. [66,67] identified 10 and nine antioxidant peptides, respectively, from Caco-2 cell monolayer permeated fractions derived from silver carp muscles hydrolyzed separately using Alcalase and papain. These hydrolysates were first subjected to GI digestion with pepsin and pancreatin and then applied to the Caco-2 model to study transepithelial transport. The peptides had lengths between seven and eight amino acid residues, with molecular weights ranging from 679.37 to 930.44 Da. While di- or tripeptides are typically absorbed efficiently via the PepT1 transporter, peptides consisting of four to nine amino acid residues may also be absorbed intact via paracellular transport or transcytosis [153]. These mechanisms could explain the transepithelial permeability observed in the studies by Wang et al. [66,67]. Notably, the peptides identified by Wang et al. [66,67] were rich in hydrophobic amino acids, including proline, methionine, glycine, and isoleucine at the N-terminus. This structural feature may have contributed to their absorption and overall potential for bioavailability, as supported by Wang et al. [135], who demonstrated that peptides with hydrophobic residues at the N-terminus are more resistant to enterocyte peptidases and exhibit higher permeability across Caco-2 cell monolayers.

Among the 10 peptides identified by Wang et al. [66], LVPVAVF (Figure 3) from the papain hydrolysate exhibited the highest ROS inhibitory capacity (ratio of 27.23%), likely due to its high proportion of hydrophobic amino acids. This observation is consistent with findings from Zhang et al. [154], who reported that peptides rich in hydrophobic amino acids, such as proline, phenylalanine, valine, and leucine, can readily penetrate the lipid bilayer of cell membranes and terminate the chain reaction of ROS production. Furthermore, although IIAPPER was initially identified as an antioxidant and anti-inflammatory peptide in edible insects by Zielińska et al. [155], it was also reported by Wang et al. [66] in silver carp hydrolysate. However, the other nine peptides identified by Wang et al. [66], including LVPVAVF, appear to be novel antioxidant peptides.

In another study by Wang et al. [67], the peptides VKVGNEF and MEAPPHI (Figure 3) demonstrated superior intracellular ROS scavenging activity and MMP preservation in human umbilical vein endothelial cells compared to seven other peptides. These properties translated into increased cell viability and reduced LDH release, highlighting their protective role against oxidative stress. Additionally, both peptides significantly enhanced the activities of endogenous antioxidant enzymes, SOD and GPx [67]. The antioxidant efficacy of these peptides is attributed to a high content of hydrophobic amino acids, including proline, valine, glycine, isoleucine, alanine, phenylalanine, and methionine, distributed throughout their sequences and also present in the terminal ends. Particularly, the phenylalanine at the C-terminus of VKVGNEF plays a critical role by breaking oxidative chains through a hydrogen atom transfer mechanism. This mechanism effectively scavenges oxygen free radicals and inhibits LDL oxidation [156]. These findings highlight the ability of VKVGNEF and MEAPPHI novel antioxidant peptides, derived from Alcalase and papain hydrolysates, respectively, to scavenge free radicals, inhibit LDL oxidation, and mitigate oxidative injury in endothelial cells [67].

To mimic the digestion of fish maw, a traditional food in Asia, Dai et al. [126] produced a hydrolysate by subjecting a crude water extract of crucian carp swim bladder to simulated GI digestion. This hydrolysate exhibited protective effects on intestinal cells, mitigating oxidative stress by reducing ROS levels and increasing antioxidant enzyme activities, as well as repressing inflammation. These findings suggest that the consumption of fish maw may provide such health benefits [126].

When examining the factors impacting bioavailability, a study revealed that the peptide MKAVCFSL (Figure 3), derived from bighead carp protein hydrolysates, exhibited notable stability during simulated GI digestion, despite some fragments, such as MKA, FSL, AVCFSL, and MKAVCF, were released in the process. LC-MS/MS analysis confirmed the majority of MKAVCFSL remained intact, demonstrating resistance to pepsin-pancreatin digestion and suggesting its high potential to arrive at the intestinal wall for absorption [113]. In H_2_O_2_-induced oxidative stress models, MKAVCFSL effectively protected Caco-2 cells by maintaining redox homeostasis, preventing oxidative damage, and sustaining antioxidant enzyme activity (Table 6) [113]. These effects can be attributed to its amino acid composition, particularly its aromatic and hydrophobic residues such as phenylalanine and methionine, which are known free radical scavengers [157]. Future investigations should also include MKAVCFSL fragments, for example, MKA, FSL, AVCFSL, and MKAVCF, and exploring their individual contributions in antioxidant activities.

### 4.6. In Vivo Evidence

Generally, the in vivo antioxidant effects of carp-derived peptides and protein hydrolysates have been less extensively explored than their in vitro effects. Several recent studies have examined the antioxidant properties of carp-derived peptide mixtures/hydrolysates, testing them in animal models such as zebrafish and rats over 14–44 days (Table 7). Carp skin gelatin hydrolysates (CSGH) were shown to diminish MDA levels, an indicator of oxidative stress, while concurrently elevating glutathione reductase (GR) activity in the serum of rats [102]. The absence of substantial changes in total antioxidant status (TAS) and liver function indicators, such as aspartate aminotransferase and alanine aminotransferase, raises concerns regarding the overall effectiveness of CSGH as an antioxidant. This may be due to the use of healthy animals in the research [158], which might not have sufficiently mimicked oxidative stress conditions typically linked to antioxidant deficiencies. Furthermore, the encapsulation of CSGH in microcapsules may not have significantly enhanced its functional bioavailability. This is because CSGH is highly digestible and partially hydrolyzed, with a total digestibility of 97.02%, comparable to casein. However, a poor amino acid profile limits adequate bioavailability in a physiological context, as shown by its biological value (35.22%) and net protein utilization (33.32%). Supporting evidence for this was a slight increase in the TAS index, while the levels of serum GR and heme oxygenase-1 showed no significant changes [99].

In contrast, collagen peptides derived from silver carp demonstrated more consistent antioxidant effects. These included enhanced activities of antioxidant enzymes, such as SOD, CAT, and GPx, as well as decreased MDA levels in both the blood and skin tissue of mice [71]. These collagen peptides, particularly those with high antioxidant activity, exhibited higher efficacy than well-known antioxidants, such as tea polyphenols and casein peptides. While these findings are promising, the study did not comprehensively investigate the mechanisms underlying the antioxidant effects. In addition, the study duration was relatively short, only 14 days [71].

The results from the zebrafish model are noteworthy, as the administration of carp protein hydrolysate significantly increased gill antioxidant capacity against peroxyl radicals. Nevertheless, this did not prevent lipid peroxidation, as measured by thiobarbituric acid-reactive species (TBARS), in the gills of zebrafish fed diets supplemented with 50 g/kg and 100 g/kg hydrolysate [108]. The study also reported a reduction in brain lipid peroxidation but was unable to measure brain antioxidant capacity owing to the limited availability of the zebrafish brain tissue. The authors asserted that the hydrolysate could cross the blood–brain barrier; however, insufficient evidence was provided to substantiate this claim. Future research should evaluate both the effects of the hydrolysates on the brain antioxidant capacity in zebrafish and their ability to penetrate the blood–brain barrier. This would provide a clearer understanding of the neuroprotective potential of the carp protein hydrolysates.

## 5. Potential Applications in Food Preservation and Quality Improvements

Carp-derived peptides and hydrolysates have been explored for their potential applications in food systems, with their role as natural preservatives being the most studied functionality. They have been tested in a wide variety of food matrices, including sardine mince [127], blueberry [159], frozen surimi [114], fresh noodles [80], and fish filet [62,160]. Typically, carp peptides and hydrolysates are either incorporated directly into food model systems or integrated into food packaging films. In some cases, various additives, including curcumin extract and lemongrass essential oil, were introduced to further enhance the food preservation potential of carp-derived peptides and hydrolysates [161]. A recent active packaging innovation by Malekkolaei et al. [160] employed nano-encapsulated crucian carp hydrolysate in edible chitosan-fucoidan coatings, which effectively enhanced the shelf life of beluga sturgeon (*Huso huso*) filets. Beyond direct incorporation and encapsulation, Maillard reaction modification with xylo-oligosaccharides, a prebiotic oligosaccharide, was reported to improve the sensory characteristics (fishy smell and bitterness) and antioxidant properties of silver carp hydrolysates [162].

### 5.1. Antioxidant Preservation of Food Quality

Carp peptides and hydrolysates preserve food quality through antioxidant effects, primarily by mitigating protein and lipid oxidation. In the assessment for antioxidant/radical scavenging potential, 2,2-diphenyl-l-picrylhydrazyl (DPPH), ABTS, and Ferric Reducing Antioxidant Power (FRAP) assays are the methods most commonly used by most research groups [61,70,159]. During food storage, freeze–thaw cycles can induce both protein oxidation and degradation, negatively affecting the quality of stored food. Several test parameters are frequently used to assess the food preservative potential of carp-derived hydrolysate. To measure protein oxidation, researchers evaluate the formation of carbonyl groups and disulfide bonds. Protein degradation is assessed by the loss of calcium-transporting ATPase (Ca^2+^-ATPase) activity and the formation of Total Volatile Basic Nitrogen (TVB-N). In many instances, carp-derived hydrolysates inhibited the formation of carbonyl groups [64] and disulfide bonds [78,114], while also reducing the loss of Ca^2+^-ATPase activity [74,78,114] and in the formation of TVB-N [80,115,163].

Besides protein oxidation, lipid oxidation in food can greatly compromise the quality of stored products. Reduced food lipid oxidation is another indicator of an effective food preservative agent. Carp-derived peptides and hydrolysates, whether incorporated directly into food systems or applied in food packaging films, have been shown to reduce free fatty acid content [78], peroxide value [78,115,124,127], and TBARS levels [62,80,115,124]. For example, Malekkolaei et al. [160] demonstrated that nano-encapsulated carp hydrolysate in chitosan-fucoidan coatings effectively maintained lipid oxidation parameters (peroxide and TBARS values) in beluga sturgeon filets within acceptable limits for human consumption during 16 days of refrigerated storage. The authors proposed that short peptides in the carp hydrolysate contributed to scavenging free radicals and reducing lipid oxidation in the beluga sturgeon filets. In addition, they suggested that the hydrolysate formed a protective layer that deterred oxygen penetration [160,164,165].

### 5.2. Functional and Antimicrobial Improvements

In the context of carp-derived protein hydrolysates, antimicrobial potential is a critical parameter investigated when assessing their effectiveness as food preservatives. Several research groups have reported that carp-derived hydrolysates have microbial growth-inhibitory activities, including anti-Listerial activity [80,115,124,166]. In a related study, a food packaging coating film containing a carp-derived hydrolysate and eight bioactive peptides (including nisin, a well-known antimicrobial peptide) was found to exert antimicrobial effects on six of the foodborne pathogens tested [167].

Carp hydrolysates have also been studied for functional properties in food systems. Among these functional properties, improved gel strength is the most widely reported characteristic, with several studies reporting on enhanced gel strength in food systems supplemented with carp hydrolysate [64,70,79,114]. Furthermore, the addition of carp hydrolysate was linked to improved water-holding capacity [74,79,80]. Several studies also reported better food texture (tensile strength and elasticity), when carp hydrolysate was added to the food system [114,117,124].

## 6. Potential Applications in Human Health

The reported antioxidant benefits of carp peptides and hydrolysates indicate significant potential for human health applications. Pre-clinical investigations have established their safety and efficacy, even at elevated dosages, with no recorded fatalities [71,99,102]. Furthermore, peptides derived from common carp skin, for example, have demonstrate enhanced antioxidant activities compared to established antioxidant peptides such as casein peptides when tested in in vivo models [99].

### 6.1. Skincare and Anti-Aging Applications

Carp peptides and hydrolysates are promising ingredients for skincare and anti-aging formulations owing to their antioxidant and anti-photoaging properties. Collagen peptides derived from silver carp skin attenuate ultraviolet radiation-induced damage in vivo by effectively decreasing skin weight, enhancing skin constituents, and mitigating oxidative stress [71]. Furthermore, collagen hydrolysates derived from common carp skeleton exhibit potent antioxidant activity, exceeding deer and chicken collagen at 2 mg/mL [168]. A clinical study on eight subjects showed that the hydrolysates improved skin hydration and reduced wrinkle depth compared to placebo [168]. Notably, these applications hold significant potential given the global demographic shift towards an increasingly aging population [169].

### 6.2. Internal Therapeutic and Nutritional Applications

Carp peptides and hydrolysates also offer potential internal applications for therapeutic and nutritional purposes. For instance, collagen hydrolysate derived from the skin of silver carp may be developed as a nutraceutical to mitigate osteoporosis. The hydrolysate has been shown to enhance the expression of Smad3, a signaling protein involved in bone formation, which subsequently enhances the production of bone matrix proteins such as type I procollagen, osteopontin, and matrix Gla protein [73]. Additionally, the potential of carp hydrolysates in sports nutrition was highlighted in a recent clinical trial conducted by Morawska-Tota et al. [170], in which 17 athletes consumed snacks fortified with carp skin gelatin hydrolysate for five weeks. Consistent with prior evidence on the safety and antioxidant efficacy of the hydrolysate [99], this intervention elevated plasma total antioxidant status to 363.9 ± 28.5 µmol/L, shifting it from medium to high antioxidant potential [170]. Collectively, these findings demonstrate the promising potential of carp-derived peptides for nutraceutical and sports nutrition applications.

## 7. Future Perspectives

Current knowledge on the production and potential applications of carp-derived antioxidant peptides and hydrolysates presents many opportunities for future research. These opportunities span across technological, biological, and sustainability areas. The following sections highlight some of the most promising directions.

### 7.1. Novel Microbial Proteases

Future research could intensify the exploration of novel microbial proteases for carp protein hydrolysis. Extremophilic bacterial proteases achieve comparable hydrolysis of carp samples and produce antioxidant hydrolysates as effectively as commercial enzymes [68,89,96]. Future studies should address the biological effects of these alternative protease-generated hydrolysates in cellular and in vivo models, to further substantiate their value as alternatives to commercial proteases. These proteases may expand the toolkit for generating peptides from carp, potentially releasing novel peptides and broadening the antioxidant functionality profiles of carp hydrolysates compared to those produced using regular commercial proteases. Combining the valorization of carp by-products with extremophilic bacterial proteases may lead to more efficient and environmentally friendly production of antioxidant peptides. This approach also aligns with the global trend in sustainable biocatalysis in food processing.

### 7.2. Advanced Extraction

Advanced extraction techniques, such as the pulsed electric field (PEF) technology, could be adapted to improve carp peptide yield and efficiency. PEF-assisted water extraction has been shown as an environmentally friendly and cost-effective method to extract antioxidants from marine fish by-products, such as gills, heads, and bones [171]. Furthermore, PEF-assisted enzymatic treatment enhances proteolysis and the yield of abalone (*Haliotis discus hannai* Ino) viscera protein hydrolysate [172]. Thus, PEF-assisted enzymatic hydrolysis of carp proteins may potentially increase the yield of antioxidant peptides and improve their extraction efficiency, which warrants further research.

### 7.3. In Silico and Artificial Intelligence-Driven Discovery

Future studies may also integrate in silico tools, such as the BIOPEP-UWM database (https://biochemia.uwm.edu.pl/biopep/start_biopep.php) [173] and AnOxPePred (https://services.healthtech.dtu.dk/services/AnOxPePred-1.0/) [174], into their wet-lab research to expedite the screening for peptide candidates from the massive pools of peptide sequences identified from carp hydrolysates. Furthermore, incorporation of artificial intelligence, particularly deep learning, may significantly enhance the efficiency of molecular docking in the identification of potential antioxidant peptides [175], making this a crucial research direction to explore in the discovery of carp antioxidant peptides.

### 7.4. Human Health Considerations and Bioavailability

To more fully unlock the antioxidant potential of carp-derived peptides and hydrolysates across both health and food applications, there is a need for a more comprehensive understanding of how carp-derived peptides are absorbed, metabolized, and distributed in the human body. Key questions that remain to be addressed include:

• How do carp-derived peptides with proven cellular antioxidant activity perform in vivo with respect to GI digestion resistance, bioavailability, and systemic potency?
• Can these peptides exert in vivo antioxidant effects by downregulating pro-oxidative enzymes, such as lipoxygenase, myeloperoxidase, and xanthine oxidase?
• To what extent are carp-derived antioxidant peptides multifunctional, modulating other non-antioxidant physiological pathways in vivo?
• How feasible is the targeted delivery of these peptides to specific organs or tissues in vivo?
• What are the long-term in vivo effects of consuming carp-derived antioxidant peptides?
• What are the in vivo allergenic and toxicological risks of carp-derived peptides? Given carp’s known allergenicity, primarily due to parvalbumin [176], the allergenic potential of carp-derived antioxidant peptides must be carefully evaluated. Although enzymatic hydrolysis can reduce allergenic epitopes, a comprehensive allergenicity risk assessment, including immunoassays such as ELISA [177], is essential before their use in commercial health or food products.

Rigorous in vivo studies, including animal models and well-designed human trials, are necessary to address these questions. Carp-derived peptides, whose antioxidant effects are well-documented in cellular models, could be prioritized to accelerate the process of bridging the gap between cellular evidence and in vivo studies. On the other hand, the findings of Hu et al. [137] identified FTGML as a promising candidate for skin whitening and hyperpigmentation therapy. However, their study did not address whether prolonged treatment might disrupt cellular homeostasis or lead to unintended consequences, such as oxidative stress rebound. FTGML can plausibly be administered to the body via either oral or topical routes. Future research could use in vivo models, including clinical trials, to evaluate both the feasibility of oral and topical applications. Oral administration requires assessment of GI stability and bioavailability following intestinal absorption. Conversely, while topical application bypasses the risk of GI degradation, it would necessitate an assessment of skin penetration and topical integrity. Further research is warranted to evaluate the long-term safety and efficacy of this peptide to substantiate its potential as an anti-melanogenic agent.

### 7.5. Food Application Challenges and Preservation Potential

While studies suggest carp-derived peptides and hydrolysates are effective as natural food preservatives, several key questions remain:

• Most studies have focused on examining their food preservative potential at low temperatures. It remains unclear whether these peptides and hydrolysates will exhibit comparable preservative efficacy on food products stored at non-refrigerated temperatures. Future research should address this question, as effective preservation without refrigeration would broaden the range of food products that can be preserved using carp peptides and hydrolysates.
• There is a lack of systematic comparison of the food preservation capacities of carp peptides and hydrolysates versus those from other fish species. Such comparisons could clarify whether carp have a competitive edge over other fishes as sources of food preservatives.
• Most research has focused on assessing the food preservative potential of carp-derived hydrolysates, rather than specific, purified carp-derived peptides. A more in-depth characterization of the peptide profile of an effective carp-derived hydrolysate is necessary to identify the specific peptides responsible for the observed preservative effects. This is also crucial for the future development of standardized, commercially viable, carp-based preservatives with consistent, predictable preservative activity.

### 7.6. Impact of Aquaculture Practices and Feed Regimes

Lastly, there are limited comparisons of antioxidant peptides and hydrolysates derived from carp cultured under different farming practices and fish feed regimes. Future research is necessary to investigate the effects of aquaculture conditions on the efficacy and yield of antioxidant peptides and hydrolysates from carp. Such comparative studies could provide better-informed strategies for producing carp as a source of high-quality antioxidant peptides and hydrolysate. Strategic collaboration between the aquaculture industry and research institutions is critical to realizing the potential of carp as a sustainable and reliable source of antioxidant peptides and hydrolysates for applications in health products, functional foods, and other industries.

## 8. Conclusions

Carp-derived antioxidant peptides and hydrolysates are a versatile resource with potential applications in human health, cosmetics, and food preservation. The high protein content of carp, especially its abundance of aromatic and hydrophobic amino acids, makes it an ideal raw material for antioxidant peptide production. Enzymatic hydrolysis of carp proteins has led to the discovery of potent antioxidant peptides and hydrolysates, which effectively protect against cellular and in vivo oxidative stress. Notably, the valorization of carp by-products has transformed low-value waste into high-value peptides and hydrolysates, promoting sustainability. In food systems, carp hydrolysates enhance oxidative stability and prolong shelf life. Health applications of carp-derived antioxidant peptides and hydrolysates encompass nutraceuticals, skin repair, and sports nutrition. Key challenges include validating bioavailability and long-term safety of these peptides and hydrolysates, in addition to optimizing their preservation efficacy under non-refrigerated conditions. Future research should also harness advanced techniques to fully realize carp’s potential. In conclusion, carp-derived antioxidant peptides and hydrolysates offer solutions as nutraceuticals and natural food preservatives, and contribute to the valorization of food industry by-products.

## Figures and Tables

**Table 1:** Global production (*thousand tons, live weight*) of five major cultured carp species [22].

	**Year**				
**Carp Species**	**2000**	**2005**	**2010**	**2020**	**2022**
Grass carp, *Ctenopharyngodon idella*	2976.5	3396.6	4213.1	5792.0	6151.6
Silver carp, *Hypophthalmichthys molitrix*	3034.7	3690.0	3972.0	4896.3	5070.0
Catla, *Labeo catla*	602.3	1317.5	2526.4	2313.4	4145.1
Common carp, *Cyprinus carpio*	2410.4	2666.3	3299.3	4169.0	4012.7
Bighead carp, *Hypophthalmichthys nobilis*	1438.9	1929.4	2513.6	3187.2	3320.3

**Table 2:** Amino acid composition of different tissues from various carp species ^1^.

Amino Acid	Bighead Carp, Dorsal [50] (g/100 g)	Bighead Carp, Belly [50] (g/100 g)	Common Carp, Muscle [43] (%)	Grass Carp Fed with Chlorella Powder, Muscle [38] (% Dry Weight)	Grass Carp, Ventral, Dorsal, and Tail White Muscles [27] (%)	Silver Carp, Ventral, Dorsal, and Tail White Muscles [27] (%)
Alanine (Ala)	0.98 ± 0.06	0.60 ± 0.03	7.72 ± 0.99	5.30 ± 0.04	6.17 ± 0.29	6.17 ± 0.29
Arginine (Arg)	1.04 ± 0.04	0.71 ± 0.02	1.22 ± 0.03	4.90 ± 0.06	1.60 ± 0.03	1.60 ± 0.03
Aspartic acid (Asp)	1.82 ± 0.11	1.04 ± 0.15	6.84 ± 0.53	9.14 ± 0.07	6.18 ± 0.13	7.01 ± 0.82
Cysteine (Cys)	0.45 ± 0.02	0.45 ± 0.11	NA	0.32 ± 0.01	1.13 ± 0.03	1.39 ± 0.04
Glutamic acid (Glu)	2.69 ± 0.13	1.54 ± 0.18	3.43 ± 1.62	NA	4.83 ± 0.06	4.83 ± 0.06
Glutamine (Gln)	NA	NA	NA	11.47 ± 0.04	NA	NA
Glycine (Gly)	0.96 ± 0.04	0.68 ± 0.00	15.83 ± 0.54	4.36 ± 0.03	NA	NA
Histidine (His)	0.54 ± 0.04	0.27 ± 0.01	1.28 ± 1.16	1.38 ± 0.01	2.01 ± 0.09	1.34 ± 0.06
Isoleucine (Ile)	0.78 ± 0.04	0.45 ± 0.04	5.74 ± 0.84	4.08 ± 0.03	7.15 ± 0.10	6.50 ± 0.05
Leucine (Leu)	1.47 ± 0.08	0.91 ± 0.06	3.75 ± 0.65	7.75 ± 0.04	NA	NA
Lysine (Lys)	1.65 ± 0.08	0.92 ± 0.13	8.73 ± 1.01	7.86 ± 0.08	6.23 ± 0.10	1.92 ± 0.06
Methionine (Met)	0.29 ± 0.01	0.08 ± 0.04	2.92 ± 1.07	2.43 ± 0.05	4.37 ± 0.17	2.48 ± 0.42
Phenylalanine (Phe)	0.84 ± 0.05	0.70 ± 0.06	3.54 ± 0.29	3.20 ± 0.02	7.41 ± 0.05	7.02 ± 0.05
Proline (Pro)	0.65 ± 0.03	0.59 ± 0.07	8.39 ± 0.55	2.30 ± 0.01	2.71 ± 0.13	2.29 ± 0.07
Serine (Ser)	0.76 ± 0.04	0.47 ± 0.04	6.39 ± 1.56	2.74 ± 0.26	NA	NA
Threonine (Thr)	0.80 ± 0.04	0.51 ± 0.01	2.60 ± 1.18	3.97 ± 0.03	1.91 ± 0.10	1.53 ± 0.08
Tryptophan (Trp)	NA	NA	2.67 ± 0.62	NA	NA	NA
Tyrosine (Tyr)	0.70 ± 0.04	0.47 ± 0.09	3.36 ± 1.68	2.41 ± 0.04	3.44 ± 0.09	2.45 ± 0.10
Valine (Val)	0.83 ± 0.05	0.52 ± 0.08	4.37 ± 0.05	4.27 ± 0.04	2.55 ± 0.00	1.59 ± 0.04

^1^ NA, data not reported in cited studies.

**Table 3:** Proteases used for producing antioxidant protein hydrolysates from various carp tissues.

Species	Part of the Fish	Proteases ^a^	References
Silver carp	Muscle	• Alcalase	[60,61,62]
		• Papain	[63]
		• Protamex	[64]
		• Alcalase • Flavourzyme	[65]
		• Alcalase • Papain • Pepsin • Trypsin	[66,67]
		• Serine protease from cold-active bacterium *Planococcus maritimus* XJ11	[68]
	Scale	• Alkaline protease	[69]
		• Alcalase • Flavourzyme • Papain	[70]
	Skin gelatin	• Alcalase • Papain • Trypsin	[71,72]
		• Bacterial collagenase • Alkaline proteinase	[73]
		• Alcalase • Flavourzyme • Papain	[74]
	Skin collagen	• Proteinase K	[75]
	Bone	• Papain	[76,77]
	Fin	• Alcalase • Neutrase • Papain • Trypsin	[78]
	By-products (head, skin, fin, scale, bone, white muscle leftover on bones, and dark muscle)	• Alcalase • Trypsin	[79]
Grass carp	Muscle	• Neutrase	[80]
		• Alkaline proteinase	[81]
		• Simulated GI digestion (pepsin + trypsin)	[82]
		• Protamex followed by Alcalase	[83]
		• Combination of Alcalase and Neutrase	[84]
		• Alkaline protease, followed by simulated GI digestion (pepsin + trypsin + chymotrypsin)	[85]
	Skin	• Alcalase	[86]
		• Protamex	[87]
		• Alcalase • Collagenase • Proteinase K • Collagenase, followed by trypsin	[88]
		• Protease EK4-1 isolated from Arctic bacterium *Mesonia algae* K4-1	[89]
	Scale	• Alkaline protease BaApr1 from the *Bacillus altitudinis* W3	[90]
	Scale gelatin	• Papain	[91]
		• Alcalase, followed by trypsin	[92]
	Swim bladder	• Combination of alkaline protease and neutral protease	[93]
	Bone	• Flavourzyme	[94]
	Intestine	• Combination of trypsin and papain	[95]
Common carp	Muscle	• Alcalase • Protease extracted from halophilic bacterial strain *Nesterenkonia* sp.	[96]
		• Protease extracted from halophilic bacterium *Halobacillus andaensis*	[97]
		• Human gastric juice • Human duodenal juice	[98]
	Skin gelatin	• Protamex	[99,100,101,102]
		• Alcalase • Flavourzyme • Neutrase • Protamex	[103]
	Scale gelatin	• Simulated GI digestion (pepsin + pancreatin)	[104]
	Roe	• Alcalase	[105,106]
		• Alcalase • Pepsin • Trypsin	[107]
	By-products (head, viscera, bones and skin)	• Alcalase	[108]
		• Alcalase • Protamex	[109]
	Collagen extract of by-products (heads, skins, and skeletons)	• Alcalase	[110]
	• Myofibrillar proteins • Sarcoplasmic proteins	• Human gastric juice • Human duodenal juice • Pepsin • Corolase PP	[111]
Bighead carp	Muscle	• Pepsin	[112,113]
	Gill	• Alcalase • Flavourzyme • Neutral protease • Papain	[114]
	Head	• Alcalase • Alkaline protease extracted from viscera of rainbow trout (*Oncorhynchus mykiss*)	[115]
	Skin	• Protamex	[116]
	Bone	• Alcalase • Protamex	[117]
	Myofibrillar proteins	• Pepsin	[118]
Catla	Muscle	• Alcalase • Bromelain • Flavourzyme • Protamex	[119]
	Swim bladder collagens	• Pepsin • Papain	[120]
	• Intestine • Liver	• Papain	[121]
Rohu	Viscera	• Alcalase	[122]
	Head	• Flavourzyme • Neutrase • Protamex	[123]
	Swim bladder gelatin	• Alcalase	[124,125]
Crucian carp	Swim bladder	• Simulated GI digestion (pepsin + pancreatin)	[126]
	Cooking juice	• Simulated GI digestion (pepsin + pancreatin)	[57]
• Catla • Rohu • Mrigal	Muscle	• Flavourzyme	[127]
		• Papain	[127]
• Black carp • Grass carp • Silver carp • Bighead carp	Skin	• Pepsin	[128]

^a^ GI, gastrointestinal.

**Table 4:** Enzymatic hydrolysis, purification, and peptide identification strategies employed in studies where the antioxidant effects of the peptides discovered have been validated ^a^.

Species	Part of the Fish	Enzyme Used for Hydrolysis	Hydrolysis Conditions	Purification and Peptide Identification	Peptide Sequence	Validation of Peptide Activity	Reference
Grass carp	Muscle	Protamex followed by Alcalase	• Protamex: pH 8, 50 °C, 3 h • Alcalase: pH 9, 50 °C, 2 h	• GFC • Nano-LC-MS/MS	• WEPPR • WVPPR • WEAPR • WEPPK • WETPR • VEYH • VAGW • LFGY • FYYGK • APPAMW • LGGY • LLLYK	• ABTS radical scavenging activity	[83]
	Skin	Alcalase	• pH 8.5, 52 °C, 115 min	• GFC • RP-HPLC • N-terminal protein sequencing • ESI-MS	• PYSFK • GFGPEL • VGGRP	• DPPH, ABTS, and hydroxyl radical scavenging activity • Inhibition of lipid peroxidation	[86]
	Scale gelatin	Alcalase followed by trypsin	• Alcalase: pH 8, 50 °C, 4 h • Trypsin: pH 7.8, 53 °C, 50 min	• Nano-LC-MS/MS	• VGPS • YGPR • GPMGPR • SGLDGAK • FPFLR • LPAWLR • WSYALR • LLGFDNVR • LAVEAWGLK	• Hydroxyl radical scavenging activity • Fe^2+^ chelating activity	[92]
	Scale	Alkaline protease BaAprl from the *Bacillus altitudinis* W3	• pH 9.5, 50 °C, 7 h	• UF membranes (MWCO: 3 and 10 kDa) • AEC • GFC • UPLC • MS/MS	• YVQAGAAGAAAH • VKLYVLLVP • VQVLAGPVVKLY	• DPPH, ABTS, and hydroxyl radical scavenging activity • Inhibition of lipid peroxidation • Reducing power	[90]
	Swim bladder	Combination of alkaline protease and neutral protease	• pH 9, 50 °C, 4 h	• UF membranes (MWCO: 3, 5, and 100 kDa) • GFC • LC-MS/MS	• EKAPDPFRHF • GILTLKYPI • GERGPPGPM • ILTERGYSFVTT • QGPPGPPGPS • VLSLYASGRTT • DGSYNIGQR	• DPPH, ABTS, superoxide, and hydroxyl radical scavenging activity • Fe^2+^ chelating activity	[93,134]
Silver carp	Muscle	Hydrolysis with Alcalase or papain, followed by simulated GI digestion (pepsin and pancreatin)	• Alcalase: pH 8, 55 °C, 2 h • Papain: pH 7, 55 °C, 2 h • Pepsin: pH 3, 37 °C, 2 h • Pancreatin: pH 7, 37 °C, 2 h	• RP-HPLC • LC-MS/MS	From papain hydrolysate: • IIAPPER • MEAPPHI • DFDDIQKK From Alcalase hydrolysate: • AGPSIVH • VKVGNEF • FDKIEDM • LNDADIA • IPDGEKV • WETIDQL	• Protection against oxidized low-density lipoprotein-induced oxidative injury in HUVECS.	[67]
Bighead carp	Muscle	Pepsin	• pH 2, 37 °C, 5 h	• UF membranes (MWCO: 3 and 5 kDa) • GFC • Semi-preparative RP-HPLC • LC-MS/MS	• MKAVCFSL	• Protection against H_2_O_2_-induced oxidative injury in Caco-2 cells	[112,113]
Crucian carp	Cooking juice	Simulated GI digestion (pepsin and pancreatin)	• Pepsin: pH 2, 37 °C, 2 h • Pancreatin: pH 7.5, 37 °C, 2.5 h	• UF membranes (MWCO: 3 and 10 kDa) • Semi-preparative RP-HPLC • UPLC-MS/MS	• IREADIDGDGQVN • PEILPDGDHD • ASDEQDSVRL • APLEEPSSPH	• DPPH radical scavenging activity • Fe^2+^ chelating activity	[57]

^a^ ABTS, 2,2’-azino-bis(3-ethylbenzothiazoline-6-sulfonic acid); AEC, anion exchange chromatography; DPPH, 2,2-diphenyl-l-picrylhydrazyl; ESI-MS, electrospray ionization-mass spectrometry; GFC, gel filtration chromatography; GI, gastrointestinal; HUVECS, human umbilical vein endothelial cells; LC-MS/MS, liquid chromatography coupled with tandem mass spectrometry; MS/MS, tandem mass spectrometry; MWCO, molecular weight cut-off; RP-HPLC, reversed-phase high-performance liquid chromatograph y; UF, ultrafiltration; UPLC, ultra-high pressure liquid chromatography.

**Table 5:** Cellular antioxidant and other bioactivities of carp-derived protein hydrolysates ^a^.

Species	Part of the Fish	Sample Dose	Cell Model	Key Findings	Reference
Silver carp	Filets	0.625–5 mg/mL	AAPH-treated Caco-2 cells	• Antioxidant enhancement: Reduced ROS concentration	[65]
	Dorsal muscle	0.25–2 mg/mL	H_2_O_2_-treated Caco-2 cells	• Antioxidant enhancement: Reduced ROS concentration; enhanced SOD, GPx, and CAT activities • Cellular protection: Increased cell viability	[66]
	Bone	10–1500 μg/mL	UVB-irradiated L929 and HaCaT cells	• Cellular protection: Increased cell viability	[76]
			*t*-BHP-treated L929 and HaCaT cells	• Antioxidant enhancement: Reduced ROS and MDA concentrations • Cellular protection: Increased cell viability	
			LPS-treated THP-1 cells	• Anti-inflammatory activity: Reduced TNF-α and IL-1β levels	
			UVB- irradiated Mel-JuSo cells	• Anti-melanogenic activity: Reduced melanin content; inhibited tyrosinase activity	
	Skin	10–400 μg/mL	*t*-BHP-treated L929 cells	• Antioxidant enhancement: Reduced ROS concentration • Cellular protection: Increased cell viability • Tissue repair and regeneration: Promoted wound healing	[75]
	Muscle	0.25–2 mg/mL	Ox-LDL- treated HUVECs	• Antioxidant enhancement: Reduced ROS concentrations; enhanced SOD and GPx activities • Cellular protection: Increased cell viability; reduced LDH concentrations; improved MMP	[67]
	Scales	0.01–1 mg/mL	B16 cells	• Antioxidant enhancement: Higher radical scavenging activity and reducing power • Cellular protection: No cytotoxicity • Anti-melanogenic activity: Reduced tyrosinase activity	[136]
Grass carp	Skin	1 and 0.1 mg/mL	6-OHDA-treated MES 23.5 cells	• Antioxidant enhancement: Higher radical scavenging activity and reducing power • Cellular protection: Increased cell viability • Neuroprotective activity: Attenuated cytotoxicity in neuronal cells	[87]
			6-OHDA-treated SH-SY5Y cells		
	Scales	0.19–3 mg/mL	H_2_O_2_-treated HepG2 cells	• Antioxidant enhancement: Reduced ROS and MDA concentrations; enhanced SOD, CAT, and GPx activities • Cellular protection: Increased cell viability; lowered apoptosis rate; weakened DNA oxidative damage	[92]
Common carp	By-products (head, viscera, bones and skin)	0.03–1.25 mg/mL	HT-22 cells	• Antioxidant enhancement: Reduced ROS concentrations • Cellular protection: Increased cell viability	[109]
Crucian carp	Swim bladder	50–150 μg/mL	H_2_O_2_-treated IEC-6 cells	• Antioxidant enhancement: Reduced ROS and MDA concentrations; enhanced SOD, CAT, and GPx activities • Cellular protection: Increased cell viability • Anti-inflammatory effects: Reduced TNF-α, IL-6, and IL-1β levels	[126]

^a^ 6-OHDA, 6-hydroxydopamine; AAPH, 2,2′-azobis(2-amidinopropane) dihydrochloride; B16 murine, melanoma cell line; Caco-2, human intestinal carcinoma cell line; CAT, catalase; GPx, glutathione peroxidase; H_2_O_2_, hydrogen peroxide; HaCaT, human keratinocyte cell line; HepG2, human liver cancer cell line; HT-22, mouse hippocampal neuronal cell line; HUVECs, human umbilical vein endothelial cells; IEC-6, rat intestinal epithelial cell 6; IL-1β, interleukin-1 beta; IL-6, interleukin 6; L929, mouse fibroblast cell line; LDH, lactate dehydrogenase; LPS, lipopolysaccharide; MDA, malondialdehyde; Mel-JuSo, human melanoma cell line; MES 23.5, fusion of rat embryonic mesencephalic cells with murine N18TG2, neuroblastoma cells; MMP, mitochondrial transmembrane potential; ox-LDL, oxidized low-density lipoprotein; ROS, reactive oxygen species; SH-SY5Y, human neuroblastoma cells; SOD, superoxide dismutase; *t*-BHP, tert-butyl hydroperoxide; THP-1, human leukemia monocytic cell line; TNF-α, tumor necrosis factor alpha; UVB, ultraviolet B.

**Table 6:** Cellular antioxidant and other bioactivities of carp-derived peptides ^a^.

Species	Part of the Fish	Peptide	Sample Dosage	Cell Model	Key Findings	Reference
Silver carp	Steak	• GPSGPSGP • PYLIGQF • YTAYYGPIPF	100 μg/mL	H_2_O_2_-treated HepG2 cells	• Antioxidant enhancement: Reduced ROS and MDA concentrations; enhanced SOD activity	[144]
	Muscle	• ADLVHVQ • EDDIFPM • GLDDIQDR • IIAPPER • ISTSLPV • KHIPGSPF • LVPVAVF • MYPGIGDR • TGLYTAIM • PTGNPLSP	50 μg/mL	H_2_O_2_-treated Caco-2 cells	• Antioxidant enhancement: Reduced ROS concentrations	[66]
	Muscle	• AGPSIVH • DFDDIQKK • FDKIEDM • IIAPPER • IPDGEKV • LNDADIA • MEAPPHI • WETIDQL • VKVGNEF	75 μg/mL	Ox-LDL-treated HUVECs	• Antioxidant enhancement: Reduced ROS levels; enhanced SOD and GPx activities • Cellular protection: Increased cell viability; reduced LDH concentration; improved MMP	[67]
	Skin	• AOG • IO • PO	200 μM	UVA-irradiated ESF cells	• Cellular protection: Increased cell viability • Modulated signaling pathways: Suppressed c-Jun and c-Fos expression; increased TGF-β and Smad3 expression; reduced Smad7 expression • Skin regeneration and ECM support: Improved COLI and HA secretion	[72]
	Scales	• SGPAGIAGPAGPRGPAGPNGPPGKD-C(Se)	0.01–0.5 mM	B16 melanoma cells	• Antioxidant enhancement: Increased GSH concentration • Cellular protection: Low cytotoxicity • Anti-melanogenic activity: Reduced melanin content, dendrites, and melanosomes; reduced tyrosinase activity; inhibited MITF, tyrosinase, TRP-1, and TRP-2 expression • Modulated signaling pathways: Inhibited α-MSH and cAMP expression; suppressed PKA, CREB, β-catenin, GSK3β, LEF, p38, JNK, and ERK signaling	[145]
Bighead carp	Muscle	• MKAVCFSL	50 μg/mL	H_2_O_2_-treated Caco-2 cells	• Antioxidant enhancement: Increased GSH (with lowered GSSG); enhanced SOD, GPx, and GR activities • Cellular protection: Increased cell viability; reduced ROS and MDA concentrations	[112,113]
Grass carp	Scale	• FTGML	0.1–1.6 mg/mL	B16F10 cells	• Antioxidant enhancement: Reduced ROS and MDA; increased GSH; enhanced SOD, CAT, GPx activities • Cellular protection: Low cytotoxicity; promoted apoptosis • Anti-melanogenic activity: Reduced tyrosinase activity and melanin content; inhibited MITF • Modulated signaling pathways: Suppressed p-STAT3, cAMP, p-PI3K/Akt; downregulated JNK and p38 pathways	[137]

^a^ α-MSH, melanocyte-stimulating hormone; B16F10, murine melanoma cell line; c-Fos, cellular Fos proto-oncogene protein; c-Jun, cellular Jun proto-oncogene protein; Caco-2, human intestinal carcinoma cell line; cAMP, cyclic adenosine 3’, 5’-monophosphate; COLI, collagen type 1; CREB, cAMP response element-binding protein; ECM, extracellular matrix; ERK, extracellular signal-regulated kinase; ESF, embryo skin fibroblast; H_2_O_2_, hydrogen peroxide; HA, hyaluronic acid; HepG2, human liver cancer cell line; GPx, glutathione peroxidase; GR, glutathione reductase; GSH, glutathione; GSK3β, glycogen synthase kinase 3-beta; GSSG, glutathione disulfide; JNK, Jun N-terminal kinase; LDH, lactate dehydrogenase; LEF, lymphoid enhancer-binding factor; MDA, malondialdehyde; MITF, microphthalmia-associated transcription factor; MMP, mitochondrial transmembrane potential; ox-LDL, oxidized low-density lipoprotein; p38, mitogen-activated protein kinase; p-PI3K/Akt, phosphatidylinositol 3-kinase/activation of phosphorylation of protein kinase B; p-STAT3, phosphorylated signal transducer and activator of transcription 3; PKA, protein kinase A; ROS, reactive oxygen species; Smad3/7, mothers against decapentaplegic homolog 3/7; SOD, superoxide dismutase; TGF-β, transforming growth factor beta; TRP, tyrosinase-related protein; UVA, ultraviolet A.

**Table 7:** In vivo antioxidant effects of carp-derived peptide mixtures/protein hydrolysates.

Peptide Mixture/Hydrolysate	Source	Duration (days)	Dosage (g/kg)	In Vivo Model	Key Findings ^a^	References
CSGH	Skin	30	10 and 100	Healthy male Wistar rats	• Unaltered in ALT and AST levels • Decreased MDA level • Elevated GR level	[102]
Furcellaran-coated CSGH microcapsules	Skin	35	14.29	Healthy male Wistar rats	• Low ALT and AST levels • Low MDA level	[99]
Collagen peptides from silver carp	Skin	14	0.2	Female KM mice	• Increased SOD, CAT and GPx activities • Decreased MDA and protein carbonyl contents	[71]
Carp protein hydrolysate	Head, viscera, bones, and skin	44	25, 50 and 100	Zebrafish	• Gills: increased ACAP; no change in TBARS. • Brain: decreased TBARS	[108]

^a^ ACAP, antioxidant capacity against the peroxyl radical; ALT, aspartate aminotransferase; AST, alanine aminotransferase; CAT, catalase; CSGH, carp skin gelatin hydrolysate; GPx, glutathione peroxidase; GR, glutathione reductase; MDA, malondialdehyde; SOD, superoxide dismutase; TBARS, thiobarbituric acid-reactive species.

## Data Availability

Data sharing is not applicable.

## Abbreviations

The following abbreviations are used in this manuscript:

6-OHDA	6-hydroxydopamine
AAPH	2,2′-azobis(2-amidinopropane) dihydrochloride
ABTS	2,2’-azino-bis(3-ethylbenzothiazoline-6-sulfonic acid)
Ca^2+^-ATPase	calcium-transporting ATPase
CAT	catalase
DPPH	2,2-diphenyl-l-picrylhydrazyl
FRAP	Ferric Reducing Antioxidant Power
GI	gastrointestinal
GPx	glutathione peroxidase
GR	glutathione reductase
GSH	glutathione
GSSG	glutathione disulfide
H_2_O_2_	hydrogen peroxide
LDH	lactate dehydrogenase
LMW	low molecular weight
LPS	lipopolysaccharide
MDA	malondialdehyde
MMP	mitochondrial transmembrane potential
PEF	pulsed electric field
ROS	reactive oxygen species
SOD	superoxide dismutase
TAS	total antioxidant status
TBARS	thiobarbituric acid-reactive species
*t*-BHP	*tert*-butyl hydroperoxide
TVB-N	Total Volatile Basic Nitrogen
UVB	ultraviolet B
